# Expression of CNPY2 in Mouse Tissues: Quantification and Localization

**DOI:** 10.1371/journal.pone.0111370

**Published:** 2014-11-13

**Authors:** Kota Hatta, Jian Guo, Ana Ludke, Sanjiv Dhingra, Kaustabh Singh, Ming-Li Huang, Richard D. Weisel, Ren-Ke Li

**Affiliations:** 1 Division of Cardiovascular Surgery, Toronto General Research Institute, University Health Network, Toronto, Ontario, Canada; 2 Institute of Medical Sciences, University of Toronto, Toronto, Ontario, Canada; 3 Department of Gynecology and Obstetrics, First Affiliated Hospital of Harbin Medical University, Harbin, China; University of South Florida, United States of America

## Abstract

Canopy FGF signaling regulator 2 (CNPY2) is a FGF21-modulated protein containing a saposin B-type domain. *In vitro* studies have shown CNPY2 is able to enhance neurite outgrowth in neurons and stabilize the expression of low density lipoprotein receptor in macrophages and hepatocytes. However, no *in vivo* data are available on the normal expression of CNPY2 and information is lacking on which cell types express this protein in tissues. To address this, the present study examined CNPY2 expression at the mRNA and protein levels. Quantitative PCR and ELISA examination of mouse tissues showed that CNPY2 varies between organs, with the highest expression in the heart, lung and liver. Immunohistochemistry detected CNPY2 in a variety of cell types including skeletal, cardiac and smooth muscle myocytes, endothelial cells and epithelial cells. CNPY2 was also detectable in mouse blood and human and mouse uteri. These data demonstrate CNPY2 is widely distributed in tissues and suggest the protein has biological functions that have yet to be identified. Using these new observations we discuss possible functions of the protein.

## Introduction

The canopy FGF signaling regulator 2 (CNPY2) gene codes for a protein containing a predicted signal peptide, a saposin B-type domain and an endoplasmic reticulum (ER) retention sequence. The gene has previously been called HP10390, ZSIG9, TMEM4 and MSAP, with some of these aliases based on suspected characteristics (for example, ZSIG9 refers to “putative mammalian secretory peptide 9”). CNPY2 has not been extensively studied, with only three published reports assessing its function [1]–[3]. In 2003 Bornhauser *et al*. showed that CNPY2 (referred to as MSAP for MYLIP-interacting saposin-like protein) interacted with MYLIP (myosin regulatory light chain interacting protein) in a two-hybrid screen [2]. The gene has since been classified as a member of the CNPY family based on sequence. Overall, little is known about CNPY2′s function.

The CNPY family has four members, called CNPY1-4. CNPY1 has been reported to play a critical role in development, with zebrafish CNPY1 acting as a positive feedback regulator of FGF that contributes to the development of left-right body asymmetry by controlling stem cell clustering during Kupffer's vesicle organogenesis [4]. Kupffer's vesicle is an embryonic early development, ciliated organ that controls left-right development in the heart, brain and gut. CNPY1 interacts with FGFR1, and induction of this receptor by FGF8 at the midbrain-hindbrain boundary is critical for the development of the brain's tectum and cerebellum in zebrafish [5]. CNPY2 is also thought to play a role in the developing brain. During development, neurons extend projections (termed neurites) to form axons and dendrites. These neurites form synaptic connections that are critical to the development of the brain. Using neuroblastoma cells and the rat PC12 cell line as a model, CNPY2 has been shown to enhance neurite outgrowth *in vitro* [2]. Therefore, both CNPY1 and CNPY2 appear to play important roles in development. Work on CNPY3 and CNPY4 is necessary to determine if these members of the CNPY family also have functions in brain development.

CNPY2 interacts with MYLIP, an E3 ubiquitin ligase that marks its targets for lysosomal degradation. The interaction between CNPY2 with MYLIP protects targets of MYLIP from degradation, making CNPY2 an indirect modulator of protein stability. Two known targets of MYLIP are myosin regulatory light chain (MRLC), a protein involved in cytoskeletal regulation, and the low density lipoprotein receptor (LDLR) [6], [7]. The CNPY2-MYLIP-MRLC-cytoskeleton relationship is involved in CNPY2′s control of neurite outgrowth and cell mobility in neurons *in vitro* [1], [2]. Do *et al.* hypothesized that CNPY2 can also act as a regulator of LDLR expression in a similar fashion to how CNPY2 interacts with MYLIP to affect the stability of MRLC [3]. Using the mouse macrophage cell line Raw 264.7 and the human hepatocyte cell line Huh7, it was demonstrated that CNPY2 expression leads to stabilization of LDLR and prevents its MYLIP-mediated degradation [3]. Very low density lipoprotein receptor (VLDLR) and apolipoprotein E receptor 2 (ApoER2) have also been identified as targets of MYLIP degradation and suggest the CNPY2-MYLIP axis has a broad role in lipid metabolism and nervous system physiology [8]. In summary, CNPY2 protects targets of MYLIP from lysosomal degradation. The identification of new targets of MYLIP would expand our knowledge of the functional importance of CNPY2 beyond its known importance in neurite outgrowth and LDL metabolism.

CNPY2 likely has important functions that have yet to be identified in health and disease, however the understanding of this protein is preliminary and is based on limited *in vitro* studies. Cell-type specific information on CNPY2 is limited to its reported detection in cultured primary neurons and from immortalized cells lines. Data on cell populations that express this gene *in vivo* are lacking. To address this issue, our study screened tissues for CNPY2 using gene expression, protein quantification and immunohistochemistry. We sought to identify tissues and cell types that show detectable CNPY2 expression.

## Materials and Methods

### Mouse tissue collection

Use of animals was approved by the University Health Network Animal Care Committee. C57BL6 mice (n = 23) were purchased from Charles River Laboratories (St. Constant, QC) and were 8–16wks old at the time of tissue collection. Estrous cycle time points in female mice were determined by vaginal swabs [9]. Tissues were collected and frozen immediately in liquid nitrogen for mRNA and protein analyses. Tissues were processed for immunohistochemistry by fixation in 4% paraformaldehyde at 4°C for 16 h and then embedded into paraffin blocks. Replicate samples were prepared for cryosections. For frozen sectioning, fixed tissues were equilibrated stepwise to a 2∶1 mixture of 20% sucrose to Tissue-Tek O.C.T. matrix (Fisher Scientific; Ottawa, ON) and frozen [10].

### Human tissue collection

Human tissue was collected under protocols approved by the Ethics Committee of Harbin Medical University. Tissue from uterine biopsies (n = 3) and first trimester elective terminations (n = 3) were used. Tissues were fixed, processed into paraffin blocks, and used for immunohistochemistry.

### Detection of CNPY2 mRNA in mouse tissues

Quantification of *CNPY2* mRNA was performed in mouse tissues using *beta-actin* mRNA as a reference control. Total RNA was isolated from mouse tissues with Trizol reagent (Sigma Aldrich; Oakville, ON). Reverse transcription was performed using SuperScript III (Life Technologies; Burlington, ON). Quantitative PCR primers and probes were designed to either span an intron-exon junction or at least harbor one large genomic intron in between to exclude potential genomic DNA contamination. Reverse transcription was performed at 50°C for 60 min. Real-time PCR amplification was performed using a GeneAmp PCR 9600 Thermocycler (Applied Biosystems; Burlington, ON) for 40 cycles at 95°C for 15 s, 60°C for 1 min. The expression level was calculated by the comparative threshold cycle method and the relative abundance in other tissues compared to heart was expressed as 2^−[(ct of CNPY2−ct of beta−actin) in other tissues−(ct of CNPY2−ct of beta−actin) in heart]^. Primers and TaqMan probes are summarized in Table 1.

**Table 1:** Real-time PCR primers.

Gene	Forward primer	TaqMan FAM-probe	Reverse primer	Length (bp)
Mouse *CNPY2*	5′-g a a c a g a t t g a c c c t t c t a c c c c-3′	5′-c a g a t a t c a g c g g c a c c c t c a a g t t-3′	5′-t c t t c c a c a a t g c t c t c a c a c-3′	129
Mouse *beta*-actin	5′-a c c t t c t a c a a t g a g c t g c g-3′	5′-t c t g g g t c a t c t t t t c a c g g t t g g c-3′	5′-c t g g a t g g c t a c g t a c a t g g-3′	147

### CNPY2 antibody generation

The open reading frame of human CNPY2 encoding amino acids 21–182 (full sequence excluding the signal peptide) was cloned into the pET 15 b vector between the *Xho*I and *Bam*HI restriction sites (EMD Biosciences; San Diego, CA), resulting in recombinant CNPY2 with a 6× His tag at its N-terminus (His-CNPY2). The Origami *E.coli* (EMD Biosciences) host strain was used for production of recombinant human CNPY2 by induction with isopropyl beta-D-1-thiogalactopyranoside (IPTG). Recombinant CNPY2 was purified using Ni-NTA beads and used to immunize rabbits to generate rabbit polyclonal antibody, which was further purified by an affinity column (GenScript Piscataway, NJ).

To demonstrate the specificity of the antibody, immunoblotting was performed to detect the molecular weight of the recognized protein. For antibody testing, CNPY2-GFP and CNPY2-V5-His constructs were created and expressed in the cell lysate of HEK293 cells after transient transfection. Briefly, the full-length cDNA of human *CNPY2* (without its stop codon) was cloned between the *Xho*I and *Hind*III restriction sites in the pEGFPN-1 vector, resulting in a CNPY2 fusion protein tagged with GFP at the C-terminus. A CNPY2 fusion protein tagged with a smaller V5-His tag at its C-terminus was prepared by cloning the full-length human *CNPY2* cDNA (without its stop codon) in the pcDNA3.1/V5-His vector. Cloning primers are summarized in Table 2.

**Table 2:** Primers used for creation of CNPY2 expression constructs.

Plasmid vector	Forward primer	Reverse primer	Length (bp)
pcDNA3.1/V5-His and pEGFP N-1	5′-a t a CTCGAG a t g a a a g g c t g g g g t t g g-3′ *Xho* I	5′-a t a AAGCTT t a g c t c a t c a t g c g a t a t g t g c-3′ *Hind* III	546
pET 15 b	5′-a t a CTCGAG c g g a g g a g c c a g g a t c t c-3′ *Xho* I	5′-a t a GGATCC t c a t a g c t c a t c a t g c g a t a t g t-3′ *BamH* I	489

Mouse heart tissue was also probed with the newly-generated anti-CNPY2 antibody to confirm it detects native mouse CNPY2 protein at the expected molecular weight. Anti-GAPDH was used as a positive control (anti-CNPY2, 1∶1000 dilution; anti-GAPDH, 1∶10,000 dilution; secondary antibody, 1∶5000 dilution)

### Quantification of CNPY2 protein in mouse tissues using ELISA

Total proteins from tissues were analyzed by ELISA for CNPY2 quantification. Tissue protein was prepared using a homogenizer in NP-40 buffer containing 150 mM NaCl, 1% Nonidet-P40 and 50 mM Tris (pH 8.0) supplemented with a cocktail of proteinase inhibitors. For measurement of CNPY2 in mouse blood, serum was obtained after 2 h of clotting and centrifuged at 2000×g for 20 min at room temperature, and 100 µl of serum was used. To prepare the ELISA plates, 96-well plates were coated with 1 pg/ml of rabbit-α-CNPY2 antibody overnight at 4°C. Each plate was washed and coated with 50 µg of standards and sample protein in 100 µL of 50 mM carbonate buffer (pH 9.6) and incubated for 2 h at room temperature. The plates were washed and blocked for 1 h with 1% BSA in 0.1 M PBS (pH 7.2) at room temperature. Following the incubation, the plates were washed three times with 200 µL PBS containing 0.05% (v/v) Tween-20 and incubated with primary antibody (rabbit polyclonal α-CNPY2, 1∶5000, final concentration 200 ng/ml) for 2 h at room temperature. Plates were washed three times with PBS and incubated with peroxidase-labeled α-rabbit IgG secondary antibody (1∶10,000; Santa Cruz Biotechnology, Dallas, TX) for 2 h at room temperature. Wells were washed three times as described above, and developed by the addition of 100 µL of TMB substrate for 30 min. Finally, 50 µL of sulfuric acid stop solution was added to each well and the optical density of each well was measured (Biotek Instruments Winooski, VT) at 450 and 570 nm and values were correlated to the standard curve to determine protein concentration.

### Localization of CNPY2 in tissues

Paraffin embedded tissues were sectioned at 5 µm, collected on slides, dried, de-waxed in xylene and rehydrated in alcohol steps to water before antigen retrieval was performed. A battery of buffers were tested over a wide pH range to determine the optimal condition for heat-induced epitope retrieval (HIER) [11]. Buffers tested include glycine-HCl (50 mM, pH 3.6), citrate acid (10 mM, pH 6.0), EDTA (1 mM, pH 8.0) and Tris-EGTA (10 mM Tris, 0.5 mM EGTA, pH 9.0). HIER was performed with slides in solution and brought to a boil in a microwave pressure cooker (Nordic Ware; Minneapolis, MN) at 1200 W followed by continued heating at 350 W for 15 min. Of the HIER buffers used, citrate acid (10 mM, pH 6.0) produced the most sensitive detection. Following HIER, endogenous peroxidase activity was quenched by 0.3% hydrogen peroxide in 1% sodium azide in 50% methanol for 30 min [12]. Slides were washed in Tris-buffered saline (TBS), blocked for 30 min at room temperature using DAKO Serum-free blocking buffer (DAKO; Mississauga, ON) and incubated overnight at 4°C with primary antibody (rabbit α-CNPY2, 1∶200, GenScript) diluted in DAKO Antibody Diluent (DAKO). Slides were subsequently washed in TBS, incubated with secondary antibody (goat α-rabbit IgG-HRP, Santa Cruz Biotechnology) for 2 h at room temperature, washed, developed with Liquid DAB+ (DAKO), counterstained with Hematoxylin QS (VECTOR; Burlington, ON) and blued in ammonia water. Developed slides were dehydrated, cleared in xylene and mounted in Permount (Fisher Scientific; Ottawa, ON). Slides were photographed using an Eclipse Ti microscope (Nikon; Mississauga, ON) and staining intensities were scored and tabulated by modifying the conventions used by Porzionato *et al* (*i.e*.: −, not detectable; absent; +/−, barely detectable; +, weak detection; ++, moderate detection; or +++, strong detection) [13]. Staining was also visualized using fluorescent secondary antibodies. We used alpha-smooth muscle actin (clone 1A4, Sigma) and DAPI to assist visualization.

Potential loss of epitope signal from fixation, paraffin processing and HIER was estimated by comparing results with fixed frozen, unfixed frozen and fresh whole-tissue mounts. Cryosections were used without manipulation or air dried, immersed in cold acetone for 30 min, dried, and processed in a similar fashion as described above. Antigen retrieval using 0.1% Triton X-100 in TBS for 10 min or no antigen retrieval was also tested. Fresh tissues were prepared for whole-tissue immunostaining as described by others [14]. Antibody specificity was tested by the addition of recombinant CNPY2 to the primary antibody to absorb the antibody and block binding to tissue substrate. Normal rabbit serum was used as a negative control.

### Cell culture and immunofluorescence

Epithelial cell line BEAS-2B cells were grown in IMDEM with 10% FBS. Cells were fixed using 4% paraformaldehyde for 30 min and permeabilized using 0.05% Triton X-100 for 10 min. Cells were subsequently stained for CNPY2 as described above.

## Results

### CNPY2 mRNA is expressed in various tissues

**Figure 1:**
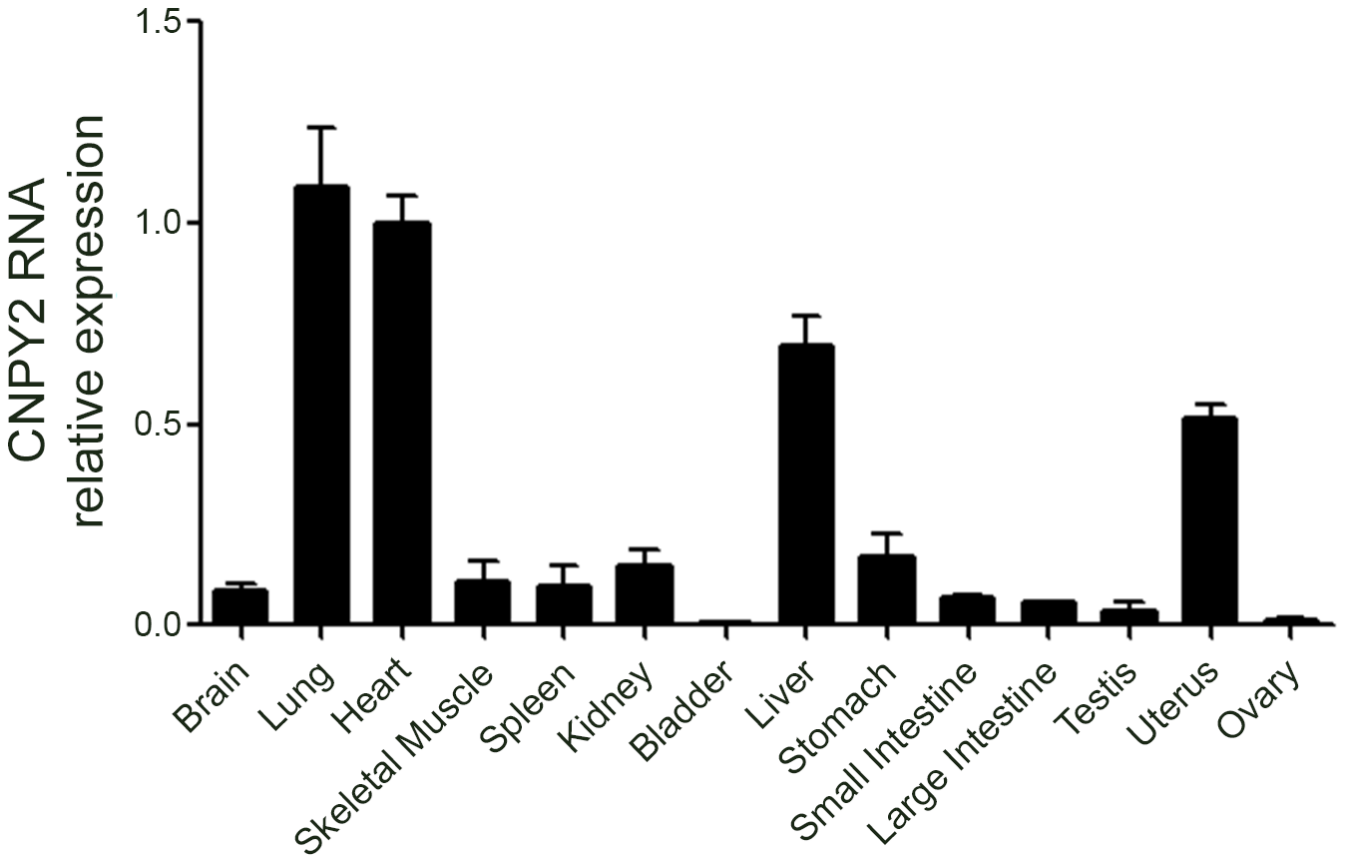
*CNPY2* mRNA is highly expressed in lung, heart and liver. Real-time PCR analysis was used to quantify *CNPY2* gene expression in mouse tissues (n = 3). The gene expression of *beta-actin* was used as a reference control to normalize expression.

Quantitative real-time PCR was used to quantify the expression level of mouse *CNPY2* in 14 tissues. *CNPY2* transcript was detectable in brain, lung, heart, skeletal muscle, spleen, kidney, bladder, liver, stomach, small intestine, large intestine, testis, uterus and ovary. As shown in Figure 1, CNPY2 was highly enriched in the lung, heart, and liver compared to other organs. The data shown in Figure 1 was normalized to *beta-actin*. When this data was repeated using *GAPDH* as a control, the expression profile was very similar (data not shown), with the highest expression levels found in lung, heart and liver.

### CNPY2 protein detection in tissue homogenates and blood

**Figure 2:**
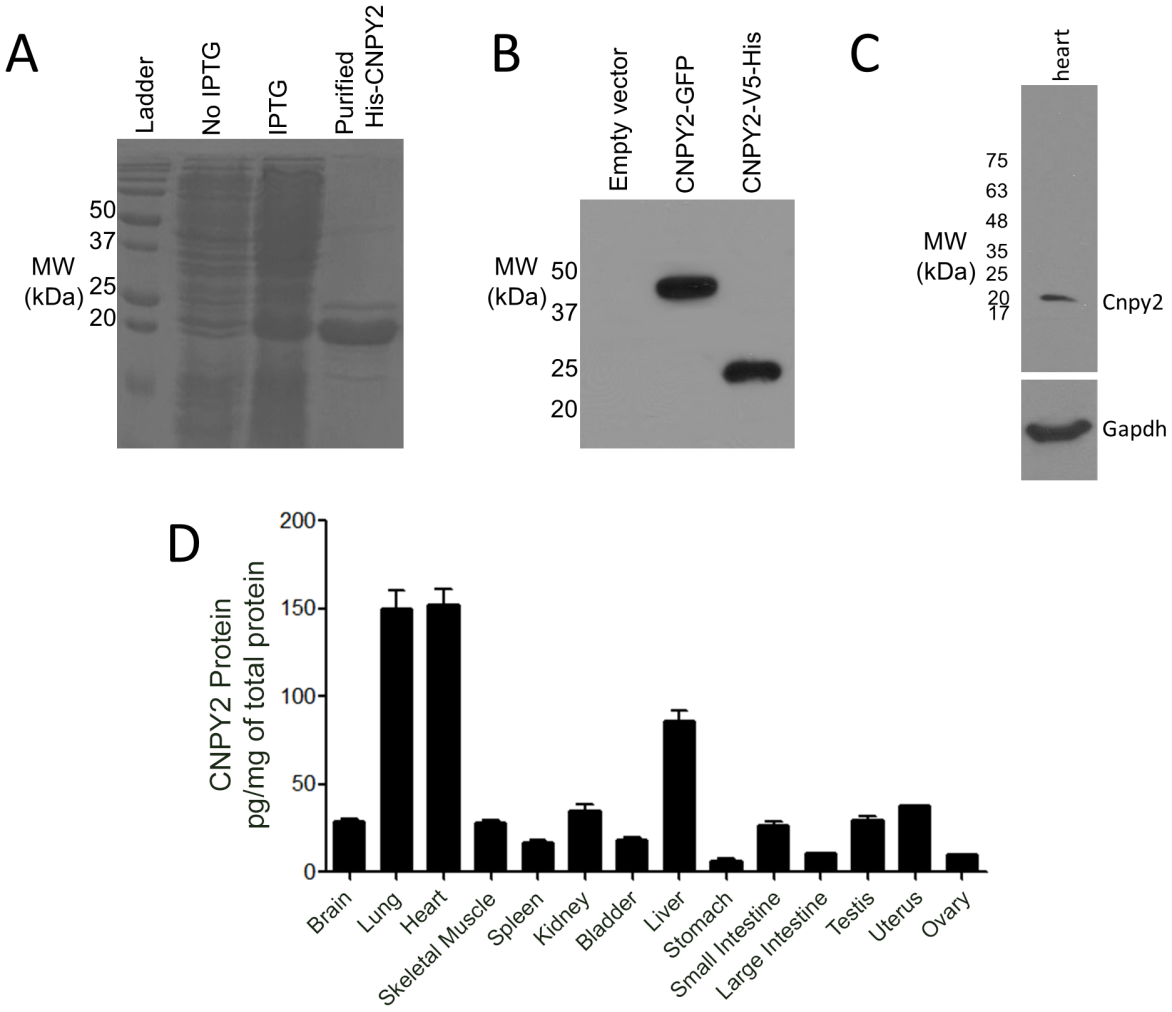
Western blotting and ELISA of CNPY2. **A.** Recombinant His-CNPY2 protein was purified from *E. coli* to generate the rabbit polyclonal antibody. The purified protein is shown on a Coomassie-stained gel. **B.** In a Western blot, the antibody was tested for detection of CNPY2 fusion proteins (CNPY2-GFP, ∼47 kDa and CNPY2-V5-His, ∼23 kDa, respectively) in the cell lysates of HEK293 cells after transient transfection. **C.** The anti-CNPY2 antibody was used to test the expression of native CNPY2 in mouse heart tissue. Anti-GAPDH served as a positive control. **D.** ELISA was performed to quantify CNPY2 protein abundance in tissues.

Recombinant CNPY2 (Figure 2A) was purified and used to generate antibody. The antibody was used to visualize CNPY2-GFP and CNPY2-V5-His on a Western blot and the predicted molecular weight bands for the respective fusion proteins were detected (Figure 2B). Native mouse CNPY2 was detected in mouse heart tissue by Western blotting (Figure 2C) and was measured in all mouse tissues examined by ELISA (Figure 2D). However, higher CNPY2 expression levels were observed in lung, heart and liver. CNPY2 was also detectable in mouse blood plasma by ELISA at a concentration of 120.0±1.4 pg/mL (n = 5).

### CNPY2 localization in tissues

**Figure 3:**
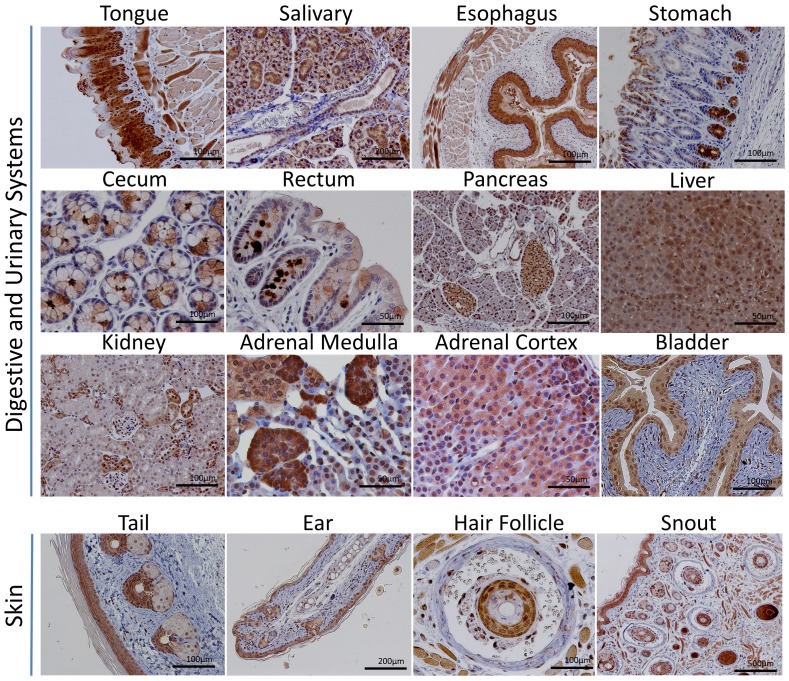
Localization of CNPY2 in digestive and urinary systems and skin tissues. CNPY2 localization was visualized by DAB (brown) and counterstained with hematoxylin.

**Figure 4:**
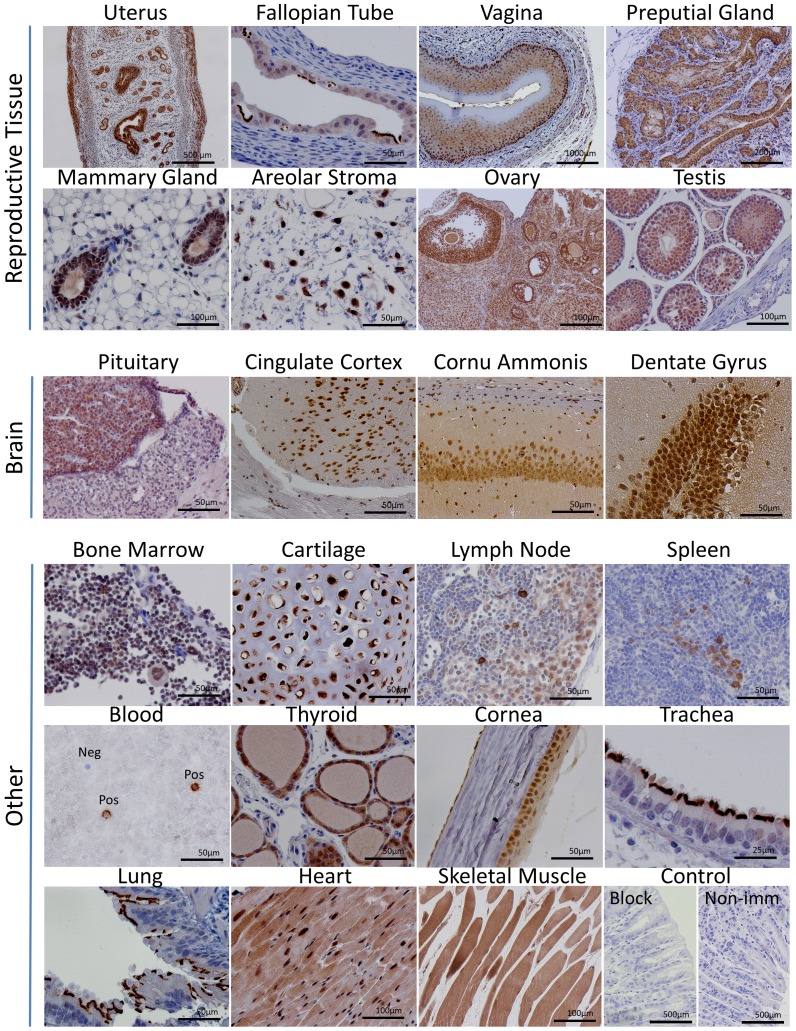
Localization of CNPY2 in reproductive tissues, brain and other sites. Immunohistochemical localization of CNPY2 was observed in a variety of tissues. The Control panel shows a peptide blocking control (Block) and a non-immunized control (Non-imm).

**Figure 5:**
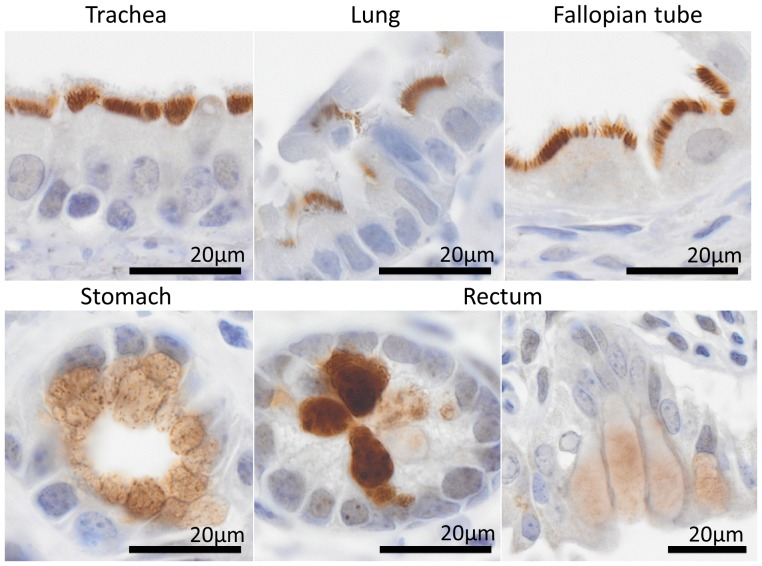
Luminal localization of CNPY2 in epithelium. High magnification images (objective: 100× oil immersion) show **CNPY**2 localization in epithelium is luminal in selected tissues.

Figure 3 shows CNPY2 staining in the digestive and urinary systems, and in the skin. Figure 4 illustrates CNPY2′s expression in reproductive and brain tissues and various others, including immune tissues such as the bone marrow and spleen as well as the heart, lung and skeletal muscle. CNPY2 was detectable in a variety of tissues (Table 3 and 4), including many types of epithelium throughout the respiratory, digestive and reproductive systems. CNPY2 could often be localized to epithelial cilia but detection was lost in cornified epithelium. CNPY2 was detected in cardiac and skeletal muscle. In the brain, neurons of the hippocampus and cerebral cortex expressed CNPY2. Bone marrow had very weak CNPY2 staining. Occasionally, rare, strongly positive-staining cells were observed in the spleen and lymph nodes. Figure 5 shows higher magnification images that demonstrate that CNPY2 is expressed on the luminal side of various epithelial tissues.

**Table 3:** Detection of CNPY2 in selected normal mouse tissues.

Tissues and organs	Cell types	Detection
Digestive system	Tongue skeletal muscle	++
	Tongue epithelium	+++
	Salivary gland epithelium	+++
	Esophagus epithelium	+++
	Stomach epithelium	++
	Duodenum epithelium	++
	Jejunum epithelium	++
	Ileum epithelium	++
	Cecum epithelium	++
	Colon epithelium	+++
	Rectum epithelium	+++
	Bile duct epithelium	++
	Liver hepatocytes	+++
	Pancreas acinar epithelium	+/−
	Abdominal cavity adipocytes	+/−
Neural system	Brain neurons	++
	Retina epithelium	+/−
	Cornea epithelium	+
	Posterior pituitary	+/−
	Pituitary anterior lobe	+
	Pituitary intermediate lobe	+/−
	Pituitary posterior lobe	−
Endocrine system	Thyroid follicular epithelium	+++
	Parathyroid principal cells	++
	Adrenal gland cortex glomerulosa	++
	Adrenal gland cortex fasiculata	++
	Adrenal gland cortex reticularis	++
	Adrenal gland medulla	+++
	Pancreas Island of Langerhans	+
	Pancreas acini cells	+/−
Uro-genital system	Kidney glomeruli	+/−
	Kidney tubular epithelium	+/−
	Bladder epithelium	++

Detection was graded: −, not detectable; +/−, barely detectable; +, weak detection; ++, moderate detection; +++, strong detection.

**Table 4:** Detection of CNPY2 in selected normal mouse tissues. Detection was graded: −, not detectable; +/−, barely detectable; +, weak detection; ++, moderate detection; +++, strong detection.

Tissues and organs	Cell types	Detection
Male reproductive system	Spermatogonias	++
	Spermatocytes	+
	Sertoli cells	+
	Leydig cells	+
	Tunica albuginea	−
Female reproductive system	Oocytes	++
(non-pregnant)	Follicular epithelium	+++
	Oviduct epithelium	++
	Uterine endometrial stroma	+
	Uterine endometrial epithelium	+++
	Uterine glandular epithelium	+++
	Preputial (clitoral) gland	+++
	Vaginal epithelium (non-cornified)	+
	Vaginal epithelium (cornified)	+/−
Respiratory system	Trachea airway epithelium	+++
	Trachea hyaline cartilage chondrocytes	+++
	Trachea seromucinous glands	+++
	Bronchial epithelium	+++
	Alveolar epithelium	++
	Endothelium	+/−
Muscular tissues	Cardiomyocytes	+
	Skeletal muscle	++
	Smooth muscle	++
Immune system	Thymus	+
	Lymph node	+/−
	Spleen red pulp	−
	Spleen white pulp	+
	Bone marrow	+/−
Skin	Epidermis epithelium (non-cornified)	++
	Epidermis epithelium (cornified)	+/−
	Sebaceous gland epithelium	+++
	Dermis fibroblasts	−
	Subcutaneous adipocytes	−
	Areolar connective tissue macrophage	++
	Subcutaneous skeletal muscle	++
	Mammary gland epithelium (non-pregnant)	+

**Figure 6:**
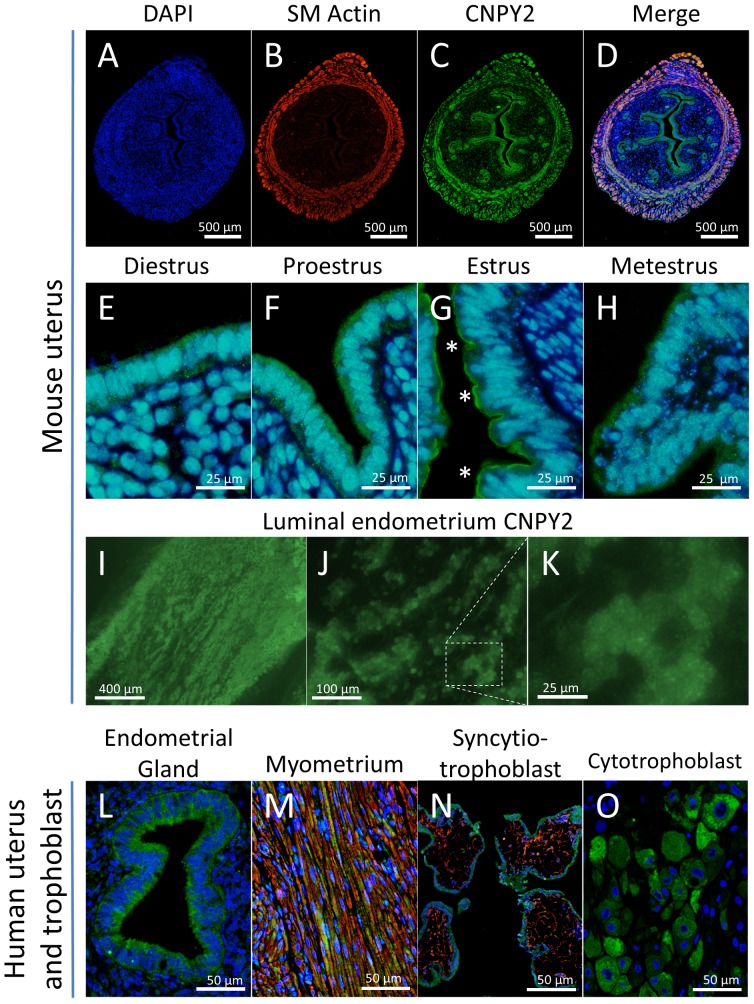
CNPY2 localization in mouse and human uteri and human trophoblast. Immunoflourescnece for **CNPY**2 was performed on mouse uteri (**A–D**) and endometrium (**E–H**) over the course of the estrous cycle. Endometrial epithelia had luminal expression at estrus (asterisks). Fresh, unfixed endometrial whole-mounts were also examined (**I–K**). Human uteri (**L, M**) and human trophoblast (**N, O**) also expressed CNPY2. Immunofluorescence shows CNPY2 (green), alpha-smooth muscle actin (red) and DAPI (blue).

In humans and mice, uterine endometrial and glandular epithelial cells as well as myometrial smooth muscle cells expressed CNPY2 (Figure 6 A–M). Additionally, human syncytiotrophoblast and cytotrophoblast stained positively for CNPY2 (Figure 6 N, O).

### CNPY2 expression in cell line BEAS-2B

**Figure 7:**
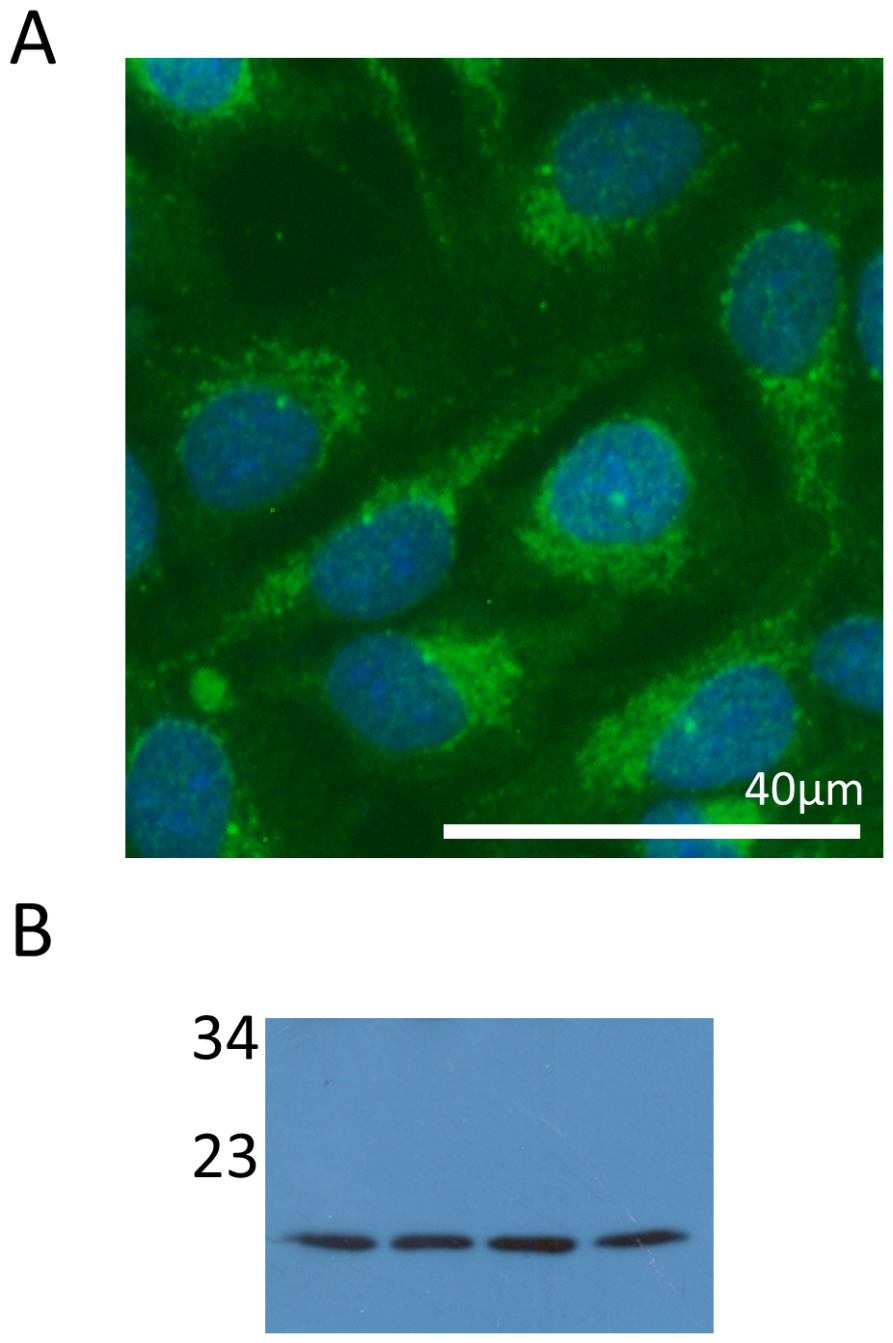
CNPY2 is detected in the epithelial cell line BEAS-2B. CNPY2 was detected in BEAS-2B cells using (**A**) immunofluorescence (CNPY2, green and DAPI, blue) and (**B**) Western blotting (numbers represent molecular weights, kDa).

Since we found prominent CNPY2 expression in epithelial cells, we decided to examine whether this protein was expressed in a human epithelial cell line. CNPY2 was detectable by immunofluorescence and Western blotting in the bronchial epithelial cell line BEAS-2B (Figure 7).

## Discussion

Here, we studied the *in vivo* expression of CNPY2 in mouse tissues and we identified several cell types that were previously not known to express CNPY2. The known functions of CNPY2 include a role in neurite outgrowth in neurons and LDLR stability in hepatocytes and macrophages. However, we detected CNPY2 in many other cell types including epithelial cells, myocytes and endothelial cells. These data suggest CNPY2 may have conserved function across different cell types as a regulator of MYLIP-mediated protein degradation. Alternatively, our data may point to new functions of this protein beyond its role as a MYLIP-associated protein.

Our observation of CNPY2 in brain and liver is consistent with previous reports that have demonstrated CNPY2 expression in C6 glioma cells and Huh7 hepatocytes [1], [3]. CNPY2 has also been studied in the mouse macrophage cell line Raw 264.7 [3]. Similarly, we detected CNPY2 in cells resembling macrophages in areolar tissue spreads. Beyond the limited work that has been dedicated to the study of CNPY2 [1]–[3], additional insights are available from microarray and proteomic screens. For example, in a proteomic comparison between human umbilical cord endothelial cells and human microvascular endothelial cells, CNPY2 protein expression was higher in the latter [15]. Consistent with these reports, we also detected CNPY2 in endothelial cells and in lung, an organ enriched with capillary endothelium. In another study, CNPY2 was among a list of genes upregulated in mammary glands during involution [16]. We similarly detected CNPY2 in mammary gland epithelium. In summary, our data are consistent with previous reports.

We demonstrated that many other cell types previously not studied for CNPY2 also express this gene and its associated protein. For example, our observations of CNPY2 in cardiac, skeletal and smooth muscle add to the list of tissues that express this protein. The detection of CNPY2 in reproductive tissues (testis, ovary, preputial gland, uterus and vagina) may lead to new postulates on the function of CNPY2 in fertility or pregnancy. Regarding the latter, in microarray screens CNPY2 was differentially expressed in diabetic pregnancies [17]. Further work is necessary to understand its function in reproduction. We also add endocrine organs to the list of tissues that express CNPY2 including the pituitary, adrenal and thyroid glands. Our data suggest CNPY2 has widespread expression and function beyond its known importance in neurons, hepatocytes and macrophages. By screening an extensive range of tissues, we are able to present comprehensive generalizations and insights on this protein. We demonstrate that CNPY2 is widely expressed in the adult organism and that its expression levels differ between organs. We also conclude CNPY2 is associated with epithelium and myocytes. We postulate the function of CNPY2 in these cell types below.

CNPY2 is an epithelium-associated protein. Others have also reported the gene in epithelium, including mammary glands [16] and placentae (composed of trophoblast, an epithelial tissue) [17]. This previous work is supported by our data and shows that CNPY2 is an epithelial-associated protein that is localized to the epithelial cells of the skin and sebaceous gland (hairy skin, ear, snout and tail), respiratory system, endocrine glands, digestive system (from the tongue to the rectum), urinary system, the eye and the reproductive system. We also detected CNPY2 in the epithelial cell line BEAS-2B. *CNPY2* transcript levels peak as the mammary gland undergoes involution [16], suggesting CNPY2 plays a role in the degradation or regeneration of epithelium. A role for epithelial cell regulation may be a shared function of the CNPY family of genes. As mentioned above, CNPY1 controls progenitor cell clustering during Kupffer's vesicle organogenesis [4]. Kupffer's vesicle is ciliated, fluid-filled epithelial organ. Luminal-basal polarization and arrangement of cilia is critical to epithelial cell clustering in Kupffer's vesicle and other epithelial organs. We observed CNPY2 localization to cilia in bronchial, oviduct and gastrointestinal epithelial cells. We postulate that CNPY2, like CNPY1, is also involved in the organization and maintenance of epithelial cells. However, further work is required. The notion of CNPY2 having a role in cytoskeletal projections like cilia may be similar to its demonstrated role in producing cellular projections during neurite outgrowth [2]. Future work on epithelial cells may reveal a conserved CNPY2-MYLIP-MRLC-cytoskeleton regulatory pathway in both neurons and epithelium.

Our study also points to the possibility that CNPY2 functions as a secreted protein. Luminal duct precipitates in the salivary gland, mammary gland lumen and the thyroid colloid all stained positively for CNPY2. These data suggest CNPY2 may be secreted. The amino acid sequence of CNPY2 has a signal peptide, suggesting it can be processed into the extracellular environment. The CNPY2 amino acid sequence also has a Saposin B-like domain [5]. Saposin B-like domain-containing proteins associate with lipids and often have extracellular functions [18]. Examples include pulmonary surfactant protein B (a lung surfactant), granulysin (a factor secreted from immune cells that disrupts the lipid membrane and lyses target cells) and acyloxyacyl hydrolase (a host defense protein that binds to lipopolysaccharide) [18]–[20]. It would not be surprising if CNPY2 likewise has an extracellular function. However, further experiments are necessary to determine the biological relevance of extracellular CNPY2.

Our observation of CNPY2 in myocytes is new, but may not be surprising because MRLC is expressed in skeletal, cardiac and smooth muscle myocytes [21]–[23]. It has been shown that increased levels of CNPY2 stabilize the expression of MYLIP targets, including MRLC [6]. MYLIP belongs to the ezrin-radixin-moesin family, a group of proteins that link cytoskeleton to membrane proteins [24]. In skeletal [21], cardiac [22] and smooth muscle cells [23], phosphorylation of MRLC plays a role in the contraction of muscle fibers. Phosphorylation of MRLC has the additional role of regulating thermogenesis in skeletal muscle [25]. Since CNPY2 controls MYLIP-mediated degradation of MRLC during muscle injury and healing, CNPY2 may play a key role in restructuring the contractile architecture of these cells. The function of CNPY2 in myocytes warrants further study.

Our study has focused only on CNPY2, while little remains known about the expression profiles of other members of the CNPY family (CNPY1, 3 and 4). Future work should compare the expression profiles and functionally characterize the cellular roles of this little-studied family of proteins. It will also be important for future studies to extend our work on CNPY2 by examining its expression profile in human tissues, beyond the placental and uterine tissue shown here.

In conclusion, CNPY2 was detected in various organs and cells. MYLIP may act on other unknown substrates, therefore additional, yet-to-be identified MYLIP substrates may also be subject to CNPY2-mediated control of protein degradation.
